# Inhibitory Activity of *Sparassis latifolia* on the Lipid Accumulation through Suppressing Adipogenesis and Activating Lipolysis in 3T3-L1 Cells

**DOI:** 10.4014/jmb.2404.04037

**Published:** 2024-07-30

**Authors:** Jeong Won Choi, Hyeok Jin Choi, Rhim Ryoo, Youngki Park, Kyoung Tae Lee, Jin Boo Jeong

**Affiliations:** 1 Department of Forest Science, Andong National University, Andong 36729, Republic of Korea; 2 Department of Forest Bioresources, Division of Forest Microbiology, National Institute of Forest Science, Suwon 16631, Republic of Korea

**Keywords:** *Sparassis latifolia*, anti-obesity, adipogenesis, lipolysis, lipophagy, thermogenesis

## Abstract

*Sparassis latifolia* (SL) has been reported to exhibit anti-obesity effects in high-fat diet animal models, yet research into its mechanisms of action remains limited. Therefore, this study aimed to elucidate the mechanisms behind the anti-obesity activity of SL's 30% ethanol extract (SL30E) using 3T3-L1 cells in an in vitro setting. SL30E effectively mitigated the accumulation of lipid droplets and triacylglycerol. SL30E downregulated PPARγ and CEBPα protein levels. The diminishment of PPARγ and C/EBPα, facilitated by SL30E, was impeded by the knockdown of β-catenin using β-catenin-specific siRNA. Furthermore, SL30E was observed to increase the protein levels of ATGL and p-HSL, while it concurrently decreased the protein levels of perilipin-1. SL30E downregulated p62/SQSTM1 protein level and upregulated LC3-II protein level. Moreover, SL30E was demonstrated to elevate the protein levels of p-AMPK and PGC-1α. The results indicate that SL30E inhibits lipid accumulation by suppressing adipogenesis and inducing lipolysis, lipophagy, and thermogenesis in 3T3-L1 cells. These observations provide potential insights into the mechanisms underlying the anti-obesity effects of SL, contributing valuable information to the existing body of knowledge.

## Introduction

Obesity is known to accelerate the onset of metabolic disorders such as type 2 diabetes [1] and cardiovascular diseases [2] and it increases the risk of mortality from cancers including esophageal, pancreatic, and renal carcinomas [3, 4]. Consequently, managing excessive body fat in response for obesity is considered one of the most significant healthcare challenges of our time [5]. Thus, numerous anti-obesity drugs have been developed to reduce body fat; however, these pharmacological interventions are commonly employed as adjunct therapies due to the tendency for weight to rebound upon cessation of treatment [6]. Furthermore, numerous anti-obesity drugs developed thus far present a significant challenge for long-term use due to serious side effects arising from long-term administration [6]. Consequently, efforts have been ongoing to explore natural anti-obesity agents that are not only effective but also safe for long-term use without adverse effects [7].

Mushrooms have been extensively utilized as food, functional food, and medicine throughout history, thereby being recognized as one of the most significant dietary supplements playing a pivotal role in human health [8]. *Sparassis latifolia* (*formerly *Sparassis crispa**), a medicinal mushroom belonging to the genus Sparassis, is recognized for its diverse pharmacological activities, including antitumor, anti-inflammatory, antiviral, antihypertensive, anti-allergic, and antidiabetic properties [9, 10]. Recent studies have reported that *Sparassis latifolia* exhibits a significant inhibitory effect on lipogenesis and enhances energy expenditure *in vivo*, thereby mitigating weight gain in mice induced by a high-fat diet [11]. This report provides direct evidence that *S. latifolia* exhibits anti-obesity activity, positioning it as a valuable natural resource for the management of obesity and obesity-mediated disorders. However, the precise mechanisms by which *S. latifolia* manifests its anti-obesity activity remain unidentified, leaving the elucidation of these mechanisms as a focal point of ongoing scientific exploration.

In this study, we report that *Sparassis latifolia* inhibits adipogenic differentiation and promotes lipolysis, autophagy, and browning in 3T3-L1 cells, thereby attenuating lipid droplet accumulation. These findings reveal the multifaceted mechanisms through which *Sparassis latifolia* exerts its anti-obesity effects. However, considering previous anti-obesity studies using animal models where *S. latifolia* demonstrated anti-obesity activity by inhibiting adipogenesis and promoting energy expenditure [11], it is likely that the inhibition of adipogenesis by SL30E could also be applicable in animal models.

## Materials and Methods

### Chemical Reagents

Dexamethasone, 3-isobutyl-1-methylxanthine (IBMX), insulin and Oil Red O were purchased from Sigma-Aldrich (USA). The primary antibodies against PPARγ (#2435), CEBPα (#8178), ATGL (#2138), HSL (#4107), p-HSL (#4137), Perilipin-1 (#9349), AMPK (#5831), p-AMPK (#2535), and β-actin (#5125), and secondary antibodies against horseradish peroxidase-linked anti-rabbit (#7074) and anti-mouse IgG (#7076) were purchased from Cell Signaling Technology, Inc. (USA). The primary antibody against PGC-1α (sc-518025) was purchased from Santa Cruz Biotechnology, Inc. (USA). Control siRNA (#6568) and β-catenin siRNA (#sc-29210) were purchased from Cell Signaling Technology, Inc. and Santa Cruz Biotechnology, Inc., respectively.

### Sample Preparation

*Sparassis latifolia* (SL) utilized in this study was provided in the form of freeze-dried, pulverized powder by the National Institute of Forest Science, Korea. SL was extracted by immersing it in 100% distilled water, 30% ethanol, 50% ethanol, and 70% ethanol, respectively, at a ratio of 20 times the volume per weight, followed by incubation at room temperature for 24 h. After 24 h, the 100% distilled water extract was centrifuged at 15,000 ×*g*, and the clear supernatant was immediately freeze-dried. Furthermore, the 30% ethanol extract, 50% ethanol extract, and 70%ethanol extract were centrifuged at 15,000 ×*g*, after which the clear supernatant had the ethanol removed via vacuum concentration before being freeze-dried. After freeze-drying, the 100% distilled water extract of SL (SL0E) was dissolved in distilled water, while the 30% ethanol extract (SL30E), the 50% ethanol extract (SL50E), and the 70% ethanol extract (SL70E) of SL were dissolved in DMSO. Each of the dissolved samples was stored at -80°C until the time of experimentation.

### Cell Culture

Due to the extensive use of 3T3-L1 cells in vitro for elucidating the anti-obesity activity and related mechanisms of action of natural compounds [12], this study also employed 3T3-L1 cells, which were purchased from the American Type Culture Collection (ATCC, USA). Cultivation of 3T3-L1 cells was conducted in a controlled CO_2_ incubator environment (37°C, 5% CO_2_) using DMEM/F-12 culture medium, which was augmented with 10%bovine calf serum (BCS) and a combination of penicillin (100 units/ml) and streptomycin (100 μg/ml).

### Differentiation of 3T3-L1 Cells

3T3-L1 cells were seeded in a 6-well plate and cultured in DMEM/F-12 medium supplemented with 10% BCS until they reached 100% confluency. Two days after reaching full confluency (designated as Day 0, D0) in 6-well culture plates, 3T3-L1 cells were cultured for 2 days in DMEM/F-12 medium containing the DMI cocktail (0.05 mM IBMX, 1 μM dexamethasone, and 10 μg/ml insulin) and 10% fetal bovine serum (FBS) (designated as Day 2, D2). Subsequently, the 3T3-L1 cells were further cultured for an additional 2 days in DMEM/F-12 medium supplemented with insulin (10 μg/ml) and 10% FBS (designated as Day 4, D4). The culture period was continued through Days 6 to 8 (designated as Day 6, D6-Day 8, D8), with medium renewal every other day.

### Transfection of Small Interference RNA (siRNA)

3T3-L1 adipocytes were seeded in 6-well plates and allowed to adhere overnight. Following initial adherence, the adipocytes underwent transfection with both a non-targeting control and β-catenin-targeted siRNAs, administered at a final concentration of 100 nM. This transfection process extended over 48 hours, utilizing the TransIT-TKO transfection reagent (Mirus, USA), executed in full accordance with the guidelines provided by the manufacturer.

### Oil Red O Staining

Following the conclusion of the treatment regimens, 3T3-L1 cells were fixed in 10% formalin at room temperature for 1 h. After fixation, the cells were washed three times with distilled water, followed by dehydration in 60% isopropanol for 5 min at room temperature. 3T3-L1 cells were then stained with Oil Red O for 20 min to enable the identification of lipid droplets. After staining, 3T3-L1 cells underwent five washes with distilled water before being observed under a light microscope at a magnification of 400× (Olympus, Japan). To quantify the lipid content, Oil Red O dye was eluted from the stained, air-dried cells using 100% isopropanol, and the absorbance of the solution was measured at 500 nm with a spectrophotometric microplate reader (Human Cop., Xma-3000PC, Republic of Korea).

### Measurement of Glycerol Contents

Upon completion of all treatment protocols, the assessment of free glycerol levels was conducted utilizing a glycerol assay kit from Cayman Chemical (Ann Arbor, USA), adhering to the guidelines provided by the manufacturer. This procedure entailed the combination of cell culture supernatant with the specifically formulated glycerol assay reagent at a ratio of 1:4, followed by a 15-min incubation at ambient temperature. Subsequent to incubation, the optical density was recorded at 540 nm with a spectrophotometric microplate reader (Human Cop., Xma-3000PC).

### Measurement of Cell Number and Viability

SL30E was administered to undifferentiated 3T3-L1 cells or to those undergoing differentiation induced by DMI and insulin from D0 to D8. At D8, the quantification of total cell count, and assessment of viability were performed utilizing a NucleoCounter NC-250 (Chemometec, Denmark), in strict adherence to the protocols specified by the manufacturer.

### Western blot Analysis

Cell lysates were prepared by incubating the cells in RIPA buffer (Boston BioProducts, USA) at 4°C for 30 min. The lysates were then centrifuged at 15,000 ×*g* at 4°C for 30 min to collect the protein supernatant. Protein concentrations were determined using the BCA protein assay kit (Thermo Fisher Scientific, USA). For electrophoresis, 30 μg of protein per well was loaded onto a 12% acrylamide gel for protein separation and run at 150 V and 400 A for 1 h. The proteins were then transferred to a nitrocellulose membrane (Thermo Fisher Scientific, Inc.) at 100 V and 300 A for 2 h. The membranes were blocked with 5% non-fat milk at room temperature for one hour, followed by overnight incubation with primary antibodies at a 1:1,000 dilution at 4°C. Secondary antibody incubation was performed at a 1:1,000 dilution at room temperature for one hour. Detection of the protein bands was achieved using ECL Prime Western Blotting Detection Reagents (GE Healthcare, UK) and visualized with a LI-COR C-DiGit Blot Scanner (LI-COR, USA). The bands were quantitatively analyzed using UN-SCAN-IT gel software version 5.1 (Silk Scientific Inc., USA). Actin was employed as a loading control for normalization in the Western blot analysis.

### Statistical Analysis

All experiments were repeated at least three times. Statistical analysis was performed using GraphPad Prism version 5.0 (GraphPad Software, Inc.) and data are presented as the mean ± standard deviation. Data were analyzed using one-way analysis of variance followed by Bonferroni’s post hoc test. *P* < 0.05 was considered to indicate a statistically significant difference.

## Results

### Effect of SL30E on the Accumulation of Lipid Droplets and Triglycerides in 3T3-L1 Cells

**Fig. 1:**
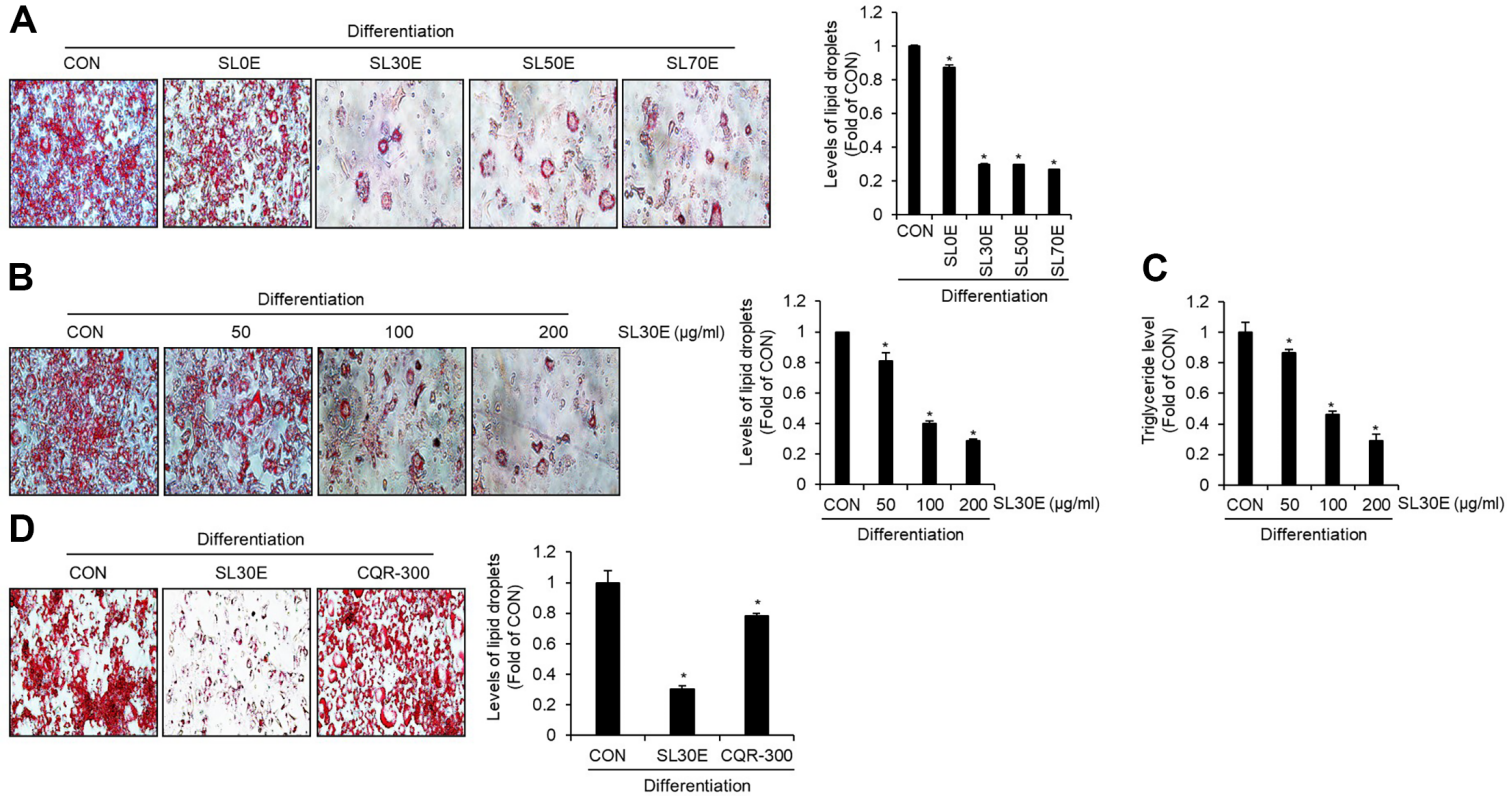
Effect of SL30E on the accumulation of lipid droplets and triglycerides in 3T3-L1 cells. 3T3-L1 cells were differentiated with DMI and insulin from D0 to D8 in the presence of the sample. At D8, the accumulation of lipid droplets and triglycerides was measured using Oil-Red O staining and ELISA Kit, respectively. (**A**) Oil-Red O staining in differentiated 3T3- L1 cells treated with SL0E, SL30E, SL50E, or SL70E. (**B**) Oil-Red O staining and (**C**) triglyceride levels in differentiated 3T3-L1 cells treated with various concentrations of SL30E. (**D**) Oil-Red O staining in differentiated 3T3-L1 cells treated with SL30E and COR-300.

To delineate the optimal extraction conditions for the anti-obesity activity of SL, this study embarked on a comparative analysis of lipid droplet accumulation inhibition of 100% aqueous extract of SL (SL0E), 30% ethanol extract of SL (SL30E), 50% ethanol extract of SL (SL50E) and 70% ethanol extract of SL (SL70E) in 3T3-L1 cells. As depicted in Fig. 1A, the SL0E exhibited a marginal inhibition of lipid droplet accumulation. In contrast SL30E, SL50E, and SL70E significantly attenuated lipid accumulation. However, no statistically significant differences were observed in the lipid droplet accumulation inhibition activity among SL30E, SL50E, and SL70E. Thus, we elected to further our investigations by focusing on SL30E. As illustrated in Fig. 1B and 1C, SL30E demonstrated a concentration-dependent reduction in both lipid droplet accumulation and triacylglycerol accumulation. In addition, we evaluated the efficacy of SL30E in inhibiting lipid accumulation in adipocytes relative to that of *Cissus quadrangularis* extract (CQR-300) [13], an agent currently utilized for anti-obesity. The results revealed that at an identical concentration of 100 μg/ml, CQR-300 inhibited lipid droplet accumulation by approximately 21.8%, whereas SL30E exhibited a markedly higher inhibition rate of approximately 69.7% (Fig. 1D).

### Effect of SL30E on the Expression Levels of Adipogenic Proteins in 3T3-L1 Cells

**Fig. 2:**
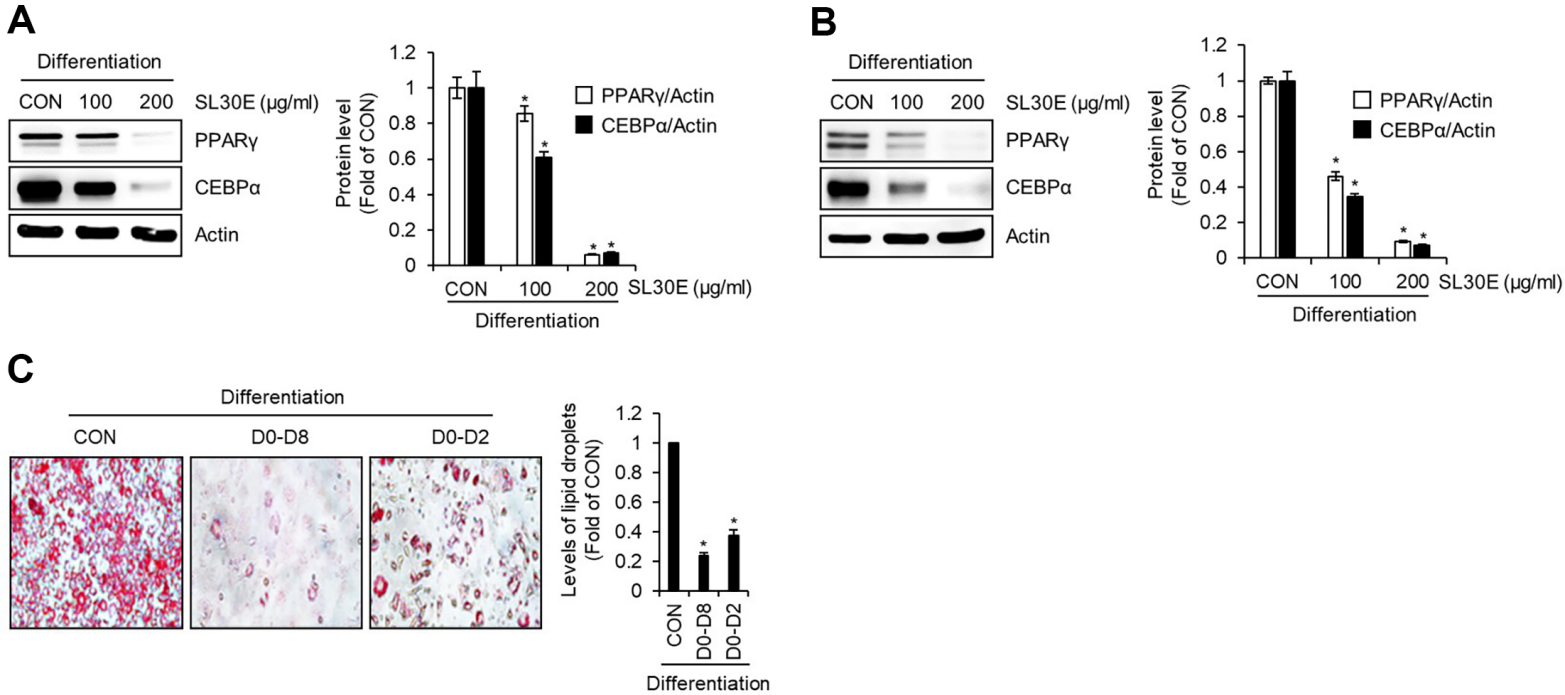
Effect of SL30E on the expression of adipogenic proteins in 3T3-L1 cells. (**A**) 3T3-L1 cells were differentiated with DMI and insulin from D0 to D8 in the presence of SL30E. At D8, the protein levels were determined by Western blot analysis. (**B**) 3T3-L1 cells were differentiated with DMI from D0 to D2 in presence of SL30E. At D2, the protein levels were determined by Western blot analysis. (**C**) 3T3-L1 cells were differentiated with DMI and insulin from D0 to D8. SL30E was treated throughout the entire period from D0 to D8 or it was treated only from D0 to D2 during the differentiation process from D0 to D8. At D8, the accumulation of lipid droplets was determined by Oil-Red O staining.

To ascertain whether the inhibitory activity of SL30E on lipid droplet accumulation was attributable to the suppression of adipogenic differentiation from preadipocytes to adipocytes, we conducted an experiment where preadipocytes were differentiated into mature adipocytes using DMI and insulin, while concurrently treating them with SL30E from D0 to D8. Subsequently, Western blot analysis was employed to examine the changes in protein levels of adipogenic markers, peroxisome proliferator-activated receptor-gamma (PPARγ) and CCATT/enhancer binding protein alpha (C/EBPα), induced by SL30E. As illustrated in Fig. 2A, at a concentration of 100 μg/ml, SL30E induced a slight decrease in the protein levels of PPARγ and C/EBPα. However, at a concentration of 200 μg/ml, SL30E resulted in a substantial reduction in the levels of these adipogenic proteins. To investigate the impact of SL30E on the protein levels of PPARγ and C/EBPα during the early stages of adipocyte differentiation, we treated preadipocytes with SL30E from D0 to D2 during differentiation induced by DMI. Western blot analysis was then conducted to assess the alterations in the protein levels of PPARγ and C/EBPα. The results, as presented in Fig. 2B, revealed that SL30E treatment at both 100 μg/ml and 200 μg/ml concentrations led to a notable decrease in the protein levels of PPARγ and C/EBPα. To ascertain whether the inhibition of adipocyte differentiation from preadipocytes by SL30E indeed contributes to the suppression of lipid droplet accumulation, we conducted an experiment where preadipocytes were differentiated into mature adipocytes using DMI and insulin from D0 to D8. During this differentiation process, SL30E was administered both throughout the entire period (D0-D8) or in the early phase (D0-D2). The accumulation of lipid droplets was then analyzed using Oil Red O staining. The findings revealed that even when SL30E was administered solely during the early phase of adipocyte differentiation (D0-D2), there was a significant reduction in lipid droplet accumulation compared to untreated cells (CON group) (Fig. 2C).

### Effect of SL30E on the β-Catenin Protein Expression in 3T3-L1 Cells

**Fig. 3:**
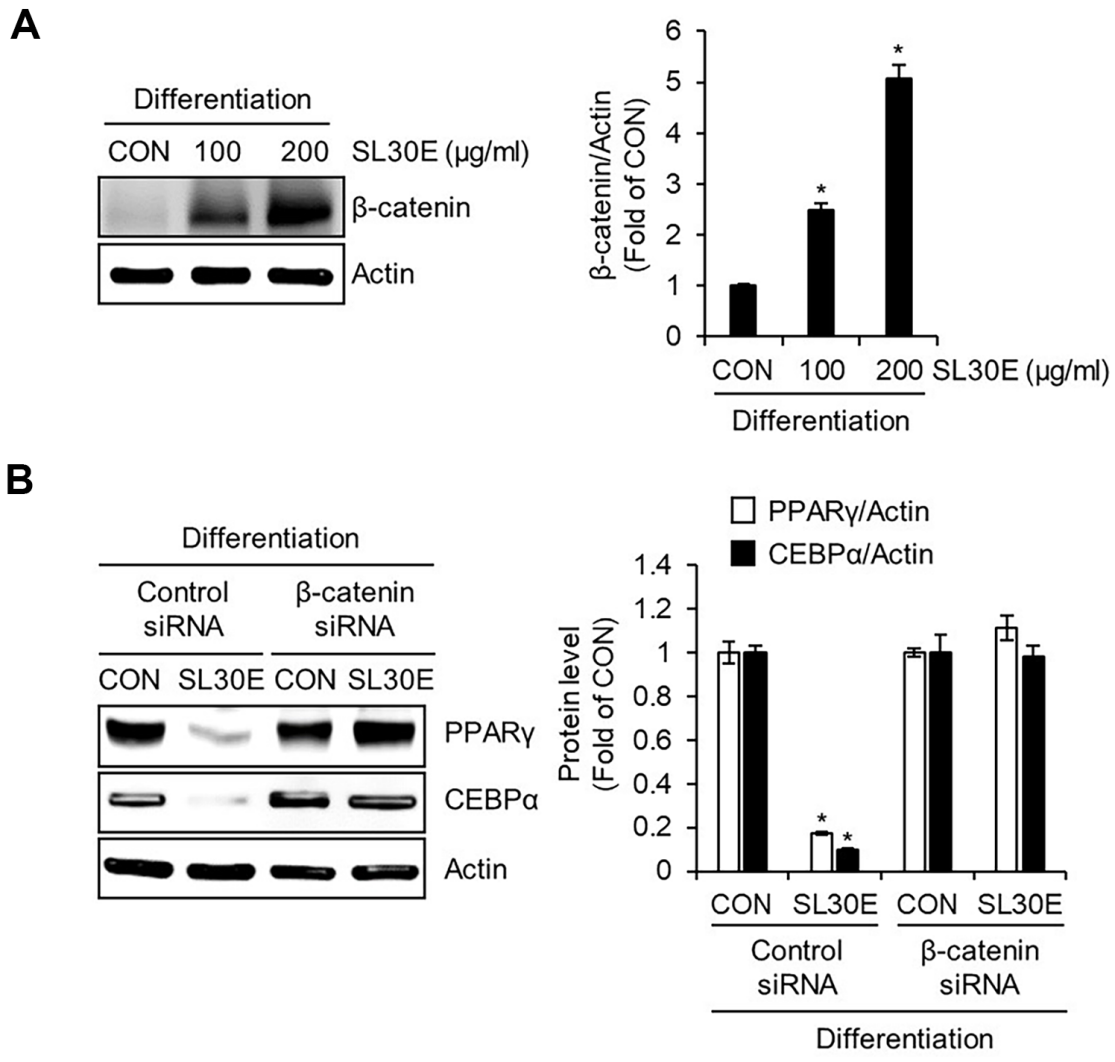
Effect of SL30E on the β-catenin protein expression in 3T3-L1 cells. (**A**) 3T3-L1 cells were differentiated with DMI from D0 to D2 in presence of SL30E. At D2, the protein levels were determined by Western blot analysis. (**B**) 3T3-L1 cells in which β-catenin was not knocked down by control-siRNA, or in which β-catenin was knocked down by β-cateninsiRNA, were differentiated with DMI from D0 to D2 in the presence of SL30E. At D2, the protein levels were determined by Western blot analysis.

β-catenin is recognized as a pivotal factor in the adipogenic differentiation process of preadipocytes, known for its role in suppressing the expression of PPARγ and C/EBPα [14]. To investigate whether the reduction in the protein levels of PPARγ and C/EBPα induced by SL30E is influenced by β-catenin, preadipocytes were differentiated using DMI from D0 to D2, during which SL30E was administered. Subsequently, Western blot analysis was conducted to assess the changes in β-catenin protein levels. The results, as depicted in Fig. 3A, indicated that in cells treated with SL30E, the protein levels of β-catenin increased in a concentration-dependent manner. To determine whether the increase in β-catenin protein levels induced by SL30E contributes to the reduction in the protein levels of PPARγ and C/EBPα, 3T3-L1 cells in which β-catenin was knocked down using β-catenin siRNA were differentiated using DMI from D0 to D2. During this differentiation process, SL30E was administered, followed by a Western blot analysis to assess changes in the protein levels of PPARγ and C/EBPα. The findings, as presented in Fig. 3B, demonstrated that in cells where β-catenin was not knocked down, the reduction in protein levels of PPARγ and C/EBPα induced by SL30E was observed. However, in cells where β-catenin was knocked down using β-catenin siRNA, the decrease in protein levels of PPARγ and C/EBPα by SL30E was not evident.

### Effect of SL30E on the Cell Growth and Cell Viability in 3T3-L1 Cells

**Fig. 4:**
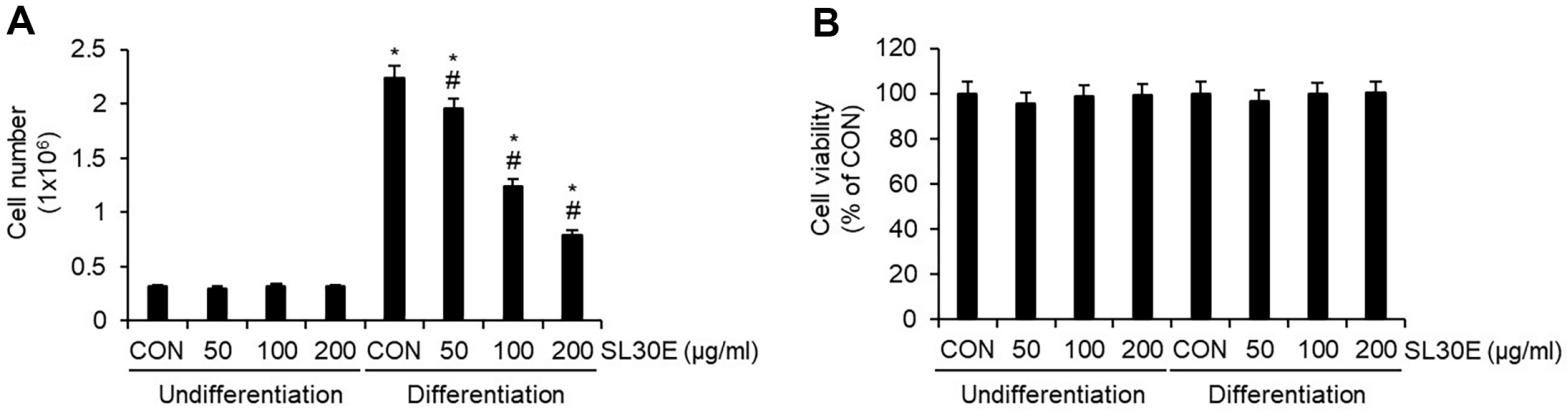
Effect of SL30E on the cell growth and cell viability in 3T3-L1 cells. SL30E was administered to undifferentiated 3T3-L1 cells or to 3T3-L1 cells undergoing differentiation induced by DMI and insulin from D0 to D8. At D8, (**A**) cell numbers and (**B**) cell viability were analyzed using a NucleoCounter NC-250 instrument.

Adipogenic differentiation from preadipocytes to adipocytes is known to be accompanied by an increase in cell number through the proliferation of adipocytes [15]. To ascertain whether SL30E inhibits the growth of adipocytes, preadipocytes were differentiated into mature adipocytes using DMI and insulin from D0 to D8, with SL30E treatment administered throughout this period. Subsequently, at D8, we analyzed the changes in the number of mature adipocytes induced by SL30E. The results indicated that in undifferentiated 3T3-L1 cells, no significant change in cell number was observed following treatment with SL30E. However, in 3T3-L1 cells differentiated using DMI and insulin, a decrease in cell number was evident as a result of SL30E treatment (Fig. 4A). However, SL30E did not affect cell viability in either undifferentiated or differentiated 3T3-L1 cells (Fig. 4B).

### Effect of SL30E on the Expression of Lipolysis, Lipophagy, and Thermogenesis-Related Proteins in 3T3-L1 Cells

**Fig. 5:**
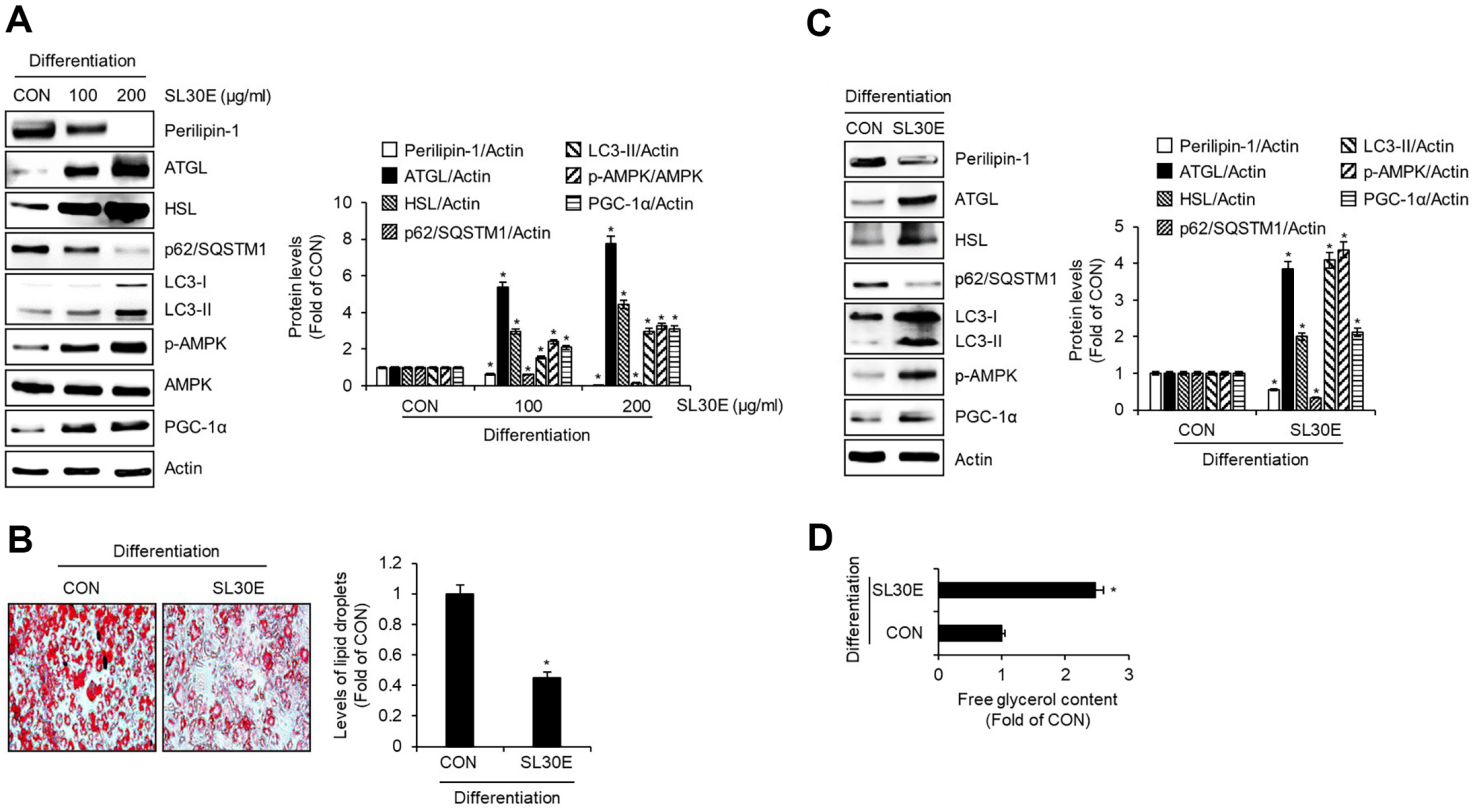
Effect of SL30E on the expression of lipolysis, lipophagy, and thermogenesis-related proteins in 3T3- L1 cells. (**A**) 3T3-L1 cells were differentiated with DMI and insulin from D0 to D8 in the presence of SL30E. At D8, the protein levels were determined by Western blot analysis. (**B-D**) 3T3-L1 cells were differentiated with DMI and insulin from D0 to D8. SL30E was treated only from D4 to D6 during the differentiation process from D0 to D8. (**B**) At D8, the accumulation of lipid droplets was measured using Oil-Red O staining. (**C**) At D8, the protein levels were determined by Western blot analysis. (**D**) At D8, free glycerol content was determined by ELISA Kit.

To determine whether SL30E impacts not only the adipogenic differentiation of preadipocytes but also influences lipolysis, autophagy, and browning in adipocytes, preadipocytes were differentiated into mature adipocytes using DMI and insulin from D0 to D8, with SL30E treatment administered throughout this period. Subsequently, at D8, Western blot analysis was conducted to assess changes in protein levels associated with lipolysis, autophagy, and browning. As depicted in Fig. 5A, SL30E led to a decrease in the protein levels of perilipin-1, which is associated with lipolysis, while it increased the protein levels of adipose triglyceride lipase (ATGL) and hormone-sensitive lipase (HSL), which is associated with lipolysis. Furthermore, in cells treated with SL30E, there was a decrease in p62/ Sequestosome-1 (SQSTM1) and an increase in microtubule-associated protein 1A/1B-light chain 3-II (LC3-II), both of which are related to lipophagy. Moreover, SL30E induced an increase in the protein levels of p-AMP-activated protein kinase (AMPK) and peroxisome proliferator-activated receptor coactivator 1-alpha (PGC-1α), which are involved in the thermogenesis. Thus, to investigate whether the activation of lipolysis, lipophagy, and thermogenesis by SL30E influences lipid accumulation inhibition, preadipocytes were differentiated into adipocytes using DMI and insulin from D0 to D8 to induce lipid accumulation. SL30E was administered from D4 to D6. Subsequently, at Day 8, changes in lipid droplet accumulation were assessed via Oil-Red O staining, alterations in protein levels were examined through Western blot analysis, and changes in free glycerol content were analyzed. As depicted in Fig. 5B, SL30E led to a reduction in lipid droplet accumulation. Furthermore, as shown in Fig. 5C, cells treated with SL30E exhibited a decrease in perilipin-1, an increase in ATGL and HSL, a decrease in p62/SQSTM1, an increase in LC3-II, and an increase in p-AMPK and PGC-1α. Additionally, as illustrated in Fig. 5D, the content of free glycerol was increased in cells treated with SL30E.

## Discussion

When energy intake exceeds energy expenditure, the surplus energy is stored in the form of lipids within adipose tissues in the body, and it is well-established that the excessive accumulation of lipids leads to obesity [16]. Thus, the ability to induce a reduction in lipid accumulation within adipose tissue may constitute one of the characteristics of a substancés anti-obesity activity. In this study, we have ascertained that SL30E significantly reduces the accumulation of lipid droplets and triglycerides in differentiated adipocytes, indicating that SL30E exhibits anti-obesity activity. Furthermore, we have determined that SL30E exhibits a higher activity in inhibiting lipid accumulation at the same concentration compared to *Cissus quadrangularis* extract (CQR-300), which is currently used as an ingredient in functional foods aimed at reducing body fat. These observations lead to the conclusion that SL30E, given its enhanced efficacy in inhibiting lipid accumulation compared to the established effects of CQR-300, holds considerable potential as a potent anti-obesity agent. Consequently, SL30E could represent a superior alternative for dietary intervention formulations targeting obesity.

Adipose tissue is primarily classified into two distinct types: white adipose tissue and brown adipose tissue. It is well-documented that the onset of obesity is associated with either an increase in the size of adipocytes (hypertrophy) within white adipose tissue or the proliferation of new adipocytes through differentiation (hyperplasia) [17]. Adipocyte hyperplasia, known as adipogenesis, refers to the complex process by which preadipocytes differentiate into mature adipocytes, culminating in an increase in the number of mature adipocytes [18]. This process is considered a key determinant in defining the number of mature adipocytes as well as the lipid mass and storage capacity accumulated within the body [19]. Therefore, the notion that inhibiting adipogenesis to regulate the size and number of adipocytes could serve as a therapeutic approach to obesity is widely accepted as a canonical principle [20]. It is well-established that PPARγ and CEBPα are pivotal transcriptional regulators acting to accelerate adipogenesis [21]. Accordingly, PPARγ and CEBPα are extensively utilized as targets for the development of anti-obesity drugs through the inhibition of adipogenesis, both in vitro and *in vivo* [22]. In this study, we observed that SL30E reduces the protein levels of PPARγ and CEBPα, and through this mechanism, inhibits adipogenic differentiation, leading to a decrease in lipid accumulation. Furthermore, SL30E was found to exclusively inhibit the increase in the number of mature adipocytes without affecting cellular viability. These findings demonstrate that SL30E can be utilized as a potential candidate for the development of anti-obesity drugs through the inhibition of adipogenesis, due to its molecular intervention in adipogenesis with minimal cytotoxic effects.

Moreover, we confirmed that SL30E increases the protein levels of β-catenin, and in cells where β-catenin is knocked down, the SL30E-induced decrease in the protein levels of PPARγ and CEBPα does not occur. Indeed, β-catenin has been reported to suppress the expression of PPARγ and CEBPα in preadipocytes, maintaining them in an undifferentiated state. Conversely, a reduction in β-catenin levels leads to an increase in the expression of PPARγ and CEBPα, thereby inducing adipogenic differentiation in preadipocytes [23]. This indicates that β-catenin plays a central role in regulating adipogenic differentiation of preadipocytes through the modulation of PPARγ and CEBPα expression. Consequently, strategies that elevate β-catenin levels could be utilized as targets for the development of compounds aimed at inhibiting adipogenesis. Therefore, our findings can serve as evidence that SL30E inhibits adipogenic differentiation of preadipocytes by decreasing the expression of PPARγ and CEBPα through the activation of β-catenin signaling.

Within the body, the regulation of lipids in adipose tissue can be achieved not only by inhibiting the initial formation of lipids but also through the removal of lipids that are in the process of accumulation or have been previously stored. If the inhibition of adipogenesis is characterized as the suppression of lipid synthesis, then lipolysis can be described as the process of breaking down lipids that are either in the midst of accumulation or have already been stored. Lipolysis refers to the sequential hydrolysis of triglycerides into diacylglycerol and subsequently into monoacylglycerol, culminating in the conversion to glycerol and fatty acids [24]. Indeed, dysfunctional lipolysis has been recognized as being closely associated with obesity [24]. HSL, responsible for the hydrolysis of triglycerides into diacylglycerol, and ATGL, which hydrolyzes diacylglycerol to monoacylglycerol, have been identified as key enzymes associated with lipolysis [25]. Recent reports indicate that dual knockout of HSL and ATGL results in a significantly more pronounced attenuation of lipolysis and a greater accumulation of lipids compared to the knockout of either ATGL or HSL alone [26]. This report suggests that the concurrent upregulation of HSL and ATGL could serve as a target for the development of anti-obesity agents aimed at inducing lipolysis. Among the critical factors associated with lipolysis is perilipin-1, which surrounds lipid droplets within adipocytes. Perilipin-1 has been reported to inhibit lipolysis and promote the formation of lipid droplets [27]. Knockout studies of perilipin-1 have shown an increase in lipolysis, leading to a reduction in the size of lipid droplets [28]. Perilipin-1 has been reported not only to restrict ATGL and HSL from lipid droplets, thereby inhibiting lipid breakdown, but also to suppress the expression of ATGL and HSL themselves [29]. In this study, we confirmed that SL30E increases the protein levels of ATGL and HSL while decreasing the protein level of perilipin-1. Additionally, we observed an increase in the content of glycerol, a hydrolysis product of triglycerides, in cells treated with SL30E. This provides evidence of SL30E's efficacy in enhancing hydrolysis triglycerides by modulating key proteins involved in lipolysis. However, a limitation of this study is the inability to elucidate the mechanisms underlying the association between the increase in ATGL and HSL and the decrease in perilipin-1 induced by SL30E. Therefore, further mechanistic studies are warranted to explore how SL30E mediates these changes in the expression levels of key proteins involved in lipolysis.

In this study, we observed a decrease in the protein levels of p62/SQSTM1 and an increase in the protein levels of LC3-II in 3T3-L1 cells treated with SL30E. The decrease in p62/SQSTM1 and the increase in LC3-II are significant indicators of the occurrence of lipophagy in adipocytes [30]. Therefore, the results of this study suggest that SL30E has the capability to induce lipophagy. Lipophagy, a form of selective autophagy, is recognized as the autophagic degradation of intracellular lipid droplets [31]. Consequently, sustained activation of lipophagy has been reported to facilitate the breakdown of lipid droplets through lipolysis, thereby inhibiting the maturation of adipocytes [32]. Furthermore, it has been reported that lipophagy plays a more significant role in the breakdown of lipid droplets than lipolysis mediated by ATGL and HSL [33]. Although this study did not analyze the impact of lipophagy inhibition on the lipid accumulation inhibitory activity of SL30E, referencing existing literature allows us to hypothesize that SL30E-mediated lipophagy may contribute to the suppression of lipid accumulation in adipocytes. Therefore, additional research is required to definitively ascertain whether SL30E induces lipophagy and how this process contributes to the inhibition of lipid accumulation.

Lastly, we also observed that SL30E increases the phosphorylation of AMPK and the protein levels of PGC-1α. These results suggest that SL30E possesses the potential to activate thermogenesis, the process of dissipating stored energy in the form of lipids as heat. The activation of AMPK leads to the stimulation of PGC-1α, thereby regulating mitochondrial biogenesis [34]. It has been reported that among natural compounds, berberine and hyperforin induce thermogenesis in white adipose tissue through the AMPK/PGC-1α signaling pathway [35, 36]. Furthermore, it is known that for the dissipation of stored energy in the form of lipids as heat through thermogenesis, the lipolysis of lipid droplets must precede [37]. Therefore, synthesizing our findings with previous reports, we propose that SL30E has the capacity to degrade triglycerides into fatty acids via lipolysis and subsequently dissipate these fatty acids as heat through thermogenesis, ultimately inhibiting lipid accumulation.

SC is known to contain a variety of bioactive compounds such as methyl orsellinate, xanthoangelol, 4-hydroxyderricin, sparoside A, hanabiratakelide A, adenosine, and 5α,6α-epoxy-(22E,24R)-ergosta-8(14),22-diene-3β,7β-diol [11]. Methyl orsellinate has shown potential in inhibiting protein tyrosine phosphatase 1B, which is a promising target for diabetes and obesity treatment [11]. Xanthoangelol and 4-hydroxyderricin are known to suppress 3T3-L1 adipocyte differentiation through the AMPK and MAPK pathways [11]. Additionally, sparoside A, hanabiratakelide A, adenosine, and 5α,6α-epoxy-(22E,24R)-ergosta-8(14),22-diene-3β,7β-diol have been found to reduce the mRNA expression of proprotein convertase subtilisin/kexin type 9, a protein that enhances the degradation of low-density lipoprotein receptors [11]. Therefore, we infer that the anti-obesity activity of SL30E is attributable to the individual or synergistic actions of these compounds. However, it is imperative to identify the specific functional components responsible for the anti-obesity mechanism of SL30E, necessitating further research in this area.

Although research into the anti-obesity activity of SL has been conducted, the investigation into the underlying mechanisms of action remains insufficient. Therefore, this study focuses on elucidating the mechanisms related to the anti-obesity effects of SL. When synthesizing the findings of this study, it can be inferred that SL30E inhibits excessive lipid accumulation in adipocytes through the suppression of adipogenesis and the promotion of lipolysis, lipophagy, and thermogenesis. These observations provide potential insights into the mechanisms underlying the anti-obesity effects of SL, contributing valuable information to the existing body of knowledge. This elucidation of SL30E's multifaceted mechanisms of action not only enhances our understanding of its therapeutic potential but also underscores the complexity of targeting obesity at the cellular level, offering a promising avenue for the development of more effective anti-obesity interventions. However, a limitation of this study is its focus solely on elucidating the mechanisms behind the anti-obesity activity of SL in vitro. Consequently, further research is necessary to determine whether SL exhibits the same mechanisms of action *in vivo*, thereby extending the applicability of our findings to physiological contexts.
